# Impact of Adverse Childhood Events on the Psychosocial Functioning of Children Affected by Parental HIV in Rural China

**DOI:** 10.3389/fpsyg.2020.617048

**Published:** 2021-01-07

**Authors:** Jordan Ezell, Sayward E. Harrison, Yanping Jiang, Xiaoming Li

**Affiliations:** ^1^Department of Psychology, University of South Carolina, Columbia, SC, United States; ^2^South Carolina SmartState Center for Healthcare Quality, University of South Carolina, Columbia, SC, United States; ^3^Department of Psychology, Wayne State University, Detroit, MI, United States; ^4^Department of Health Promotion, Education, and Behavior, Arnold School of Public Health, University of South Carolina, Columbia, SC, United States

**Keywords:** HIV, childhood adversity, childhood trauma, peer support, mental health, China

## Abstract

**Introduction**: Children affected by parental HIV are more likely than unaffected peers to experience trauma and are at-risk for negative psychological and social outcomes. This study aimed to examine the relationship between adverse childhood events and psychosocial functioning among children affected by parental HIV.

**Methods**: A total of 790 children ages 6–17 from Henan, China were enrolled in a longitudinal, randomized controlled trial of a resilience-based psychosocial intervention. At baseline, children reported on numerous psychosocial factors, including trauma exposure, symptoms of anxiety and depression, and peer social functioning. We used linear regression analysis to test the direct effect of trauma exposure on peer social functioning. We then tested whether depression and anxiety symptoms served as two potential parallel mediators in the association between trauma exposure and peer social functioning.

**Results**: Trauma exposure was significantly associated with poor peer social functioning (*β* = −0.10, *p* = 0.005) when controlling for key covariates. When depression and anxiety symptoms were added to the model, the association between trauma exposure and peer social functioning became nonsignificant. Instead, there were significant indirect effects from trauma exposure to peer social functioning *via* depression (*β* = −0.06, 95%CI[−0.09, −0.03]) and anxiety (*β* = −0.02, 95%CI[−0.04, −0.00]).

**Conclusion**: This study is among the first to link trauma exposure to peer social functioning deficits for children affected by parental HIV and demonstrates that symptoms of anxiety and depression mediate this relationship. Findings underscore the need for comprehensive psychosocial support for children affected by HIV, including screening for trauma exposure and mental health disorders.

## Introduction

Parental illness and death have profound developmental implications for children – particularly within the context of a highly-stigmatized health condition such as HIV (Cluver et al., 2013). While most individuals living with HIV are of childbearing age, mother-to-child transmission has dramatically fallen in recent decades, due to increased HIV testing among pregnant women and better access to antiretroviral therapy [United Nations Children’s Fund (UNICEF), 2015; United Nations Joint Programme on HIV/AIDS (UNAIDS), 2016]. Thus, the majority of children born to HIV-positive mothers do not acquire HIV themselves [United Nations Children’s Fund (UNICEF), 2015]. However, even healthy children of HIV-positive parents are vulnerable to a wide range of negative outcomes including orphanhood, separation from parents, HIV-related stigma, and economic hardship (Foster and Williamson, 2000; Herek et al., 2002; Sherr et al., 2008).

Children affected by parental HIV are also more likely than non-affected peers to be exposed to trauma, including caring for an ill or dying parent, parental death or separation, caregiver changes, home and school changes, and abuse or exploitation (Rotheram-Borus et al., 2001; Bauman et al., 2007; Li et al., 2009; Zhao et al., 2010). These traumatic experiences may have long-lasting, cascading effects across development, as a well-known “dose-response” relationship exists between exposure to adverse childhood experiences (ACEs) and negative outcomes across multiple domains (e.g., cognitive functioning, psychological wellbeing, behavioral health, and physical health; Anda et al., 2006; Felitti, 2009; McElroy and Hevey, 2014). Neurobiological theories of trauma highlight the impacts of hormonal functioning, brain structure and function, and genetic factors in the variation in individual response to trauma, as well as the development of psychological disorders (Heim and Nemeroff, 2001; Schnurr and Green, 2004; Heim et al., 2008; Bernaras et al., 2019). Cognitive-behavioral theories (e.g., Seligman, 1975; Beck, 1987) and social-learning theories (e.g., Bandura, 1977) highlight the role of individuals’ perceptions of events, as well as the social modeling, in the development and maintenance of non-adaptive patterns of thinking and behavior. Collectively, these altered biological and cognitive responses make it more difficult for children to cope with subsequent stress and adversity (Heim and Nemeroff, 2001; Hunt et al., 2017). Unsurprisingly then, trauma-exposed children are more likely to experience depression and anxiety than non-affected peers (Monroe and Simons, 1991; McElroy and Hevey, 2014; Nowakowski-Sims and Rowe, 2017).

Trauma exposure also has both direct and indirect effects on the social experiences of children, including peer relationships. Burgeoning literature suggests that ACEs increase risk for interpersonal difficulties, though most literature has focused on childhood sexual abuse (Poole et al., 2017). One study found significantly higher rates of peer problems among children affected by parental HIV than among non-affected peers (Doku, 2009). This is concerning because social support and strong interpersonal relationships are key protective factors for this group (Chi and Li, 2013). Social support and strong social skills are important for potentially improving children’s ability to cope with HIV-related adversity. Extended social networks (e.g., neighbors and extended families) can be a source of psychological and instrumental support for families coping with HIV, including helping meet financial, physical, and emotional needs (Betancourt et al., 2013). Effective social skills and positive peer relationships can also help children affected by parental HIV adapt to the HIV-related challenges (e.g., stigma and isolation) in school and community settings (Li et al., 2015).

Numerous studies have found that positive interactions with peers are associated with improved emotional wellbeing for children living with or affected by HIV (Funck-Brentano et al., 2005; Cluver and Gardner, 2007; Kumakech et al., 2009). Furthermore, previous research has found that children affected by HIV who report more social support display less deleterious effects from trauma exposure (Cluver et al., 2009). Thus, while a relationship between trauma experiences and social functioning among children affected by HIV has been established, additional research is needed to identify other variables that may underlie this relationship. One aspect is children’s mental health, including symptoms of anxiety and depression. A large body of literature has established that children affected by parental HIV are at greater risk for anxiety and depression (Fang et al., 2009; Guo et al., 2012; Du et al., 2015). Depression is characterized by feelings of deep sadness, lack of motivation, and social withdrawal; anxiety is characterized by persistent and excessive worry and avoidance of feared situations, including social avoidance. Thus, both disorders have major impacts for social functioning (Derdikman-Eiron et al., 2011; Verboom et al., 2014).

In summary, while the experience of parental HIV is a well-established risk factor for internalizing problems, the relationship among trauma exposure, anxiety, depression, and social functioning for children made vulnerable by parental HIV is not yet well understood. Prospective studies have shown that trauma exposure in childhood increases risk for later depression and anxiety (Scott et al., 2010). In addition, internalizing disorders in childhood have been shown to lead to poorer social functioning among children and adolescents from the general population (Verboom et al., 2014). Because of the rapid developmental changes in cognition, behavior, and social functioning across childhood and adolescence, considerations of age and developmental stage are particularly important when attempting to understand relationships between developmental context (e.g., exposure to trauma), mental health, and social functioning (Cole et al., 1998).

In order to develop effective interventions, a better understanding is needed of the relationship between trauma exposure and social functioning, as well as the impact of depression and anxiety symptoms on this relationship. Therefore, the present study aims to (1) investigate the association between trauma and peer social functioning for children affected by parental HIV, (2) determine whether symptoms of depression and anxiety mediate the relationship between trauma and peer social functioning, and (3) determine whether age moderates the indirect associations among trauma exposure, depression, anxiety, and peer social functioning. First, we hypothesize that children who report greater exposure to trauma will have more impaired social functioning. Further, we hypothesize that anxiety and depression will mediate the effects of trauma exposure on peer social functioning. We also expect that age will moderate the indirect associations, such that the negative effects of trauma on peer social functioning through depression and anxiety will be greater among older youth (i.e., ≥12 years) than among their younger counterparts. This hypothesis is informed by research that suggests that adolescence is a particularly vulnerable period for youth who have experienced early childhood trauma (Hunt et al., 2017; Nishikawa et al., 2018).

## Materials and Methods

The current study took place in Henan, China, the site of an HIV outbreak driven by commercial blood and plasma collection practices in the 1990s (Deng et al., 2008). Many residents of the rural region attempted to supplement their incomes through blood and plasma donation; collection agencies – through a variety of unhygienic practices – rapidly infected large numbers of residents with HIV, resulting in HIV prevalence estimates ranging from 10 to 60% across local villages (Wu et al., 2001; China Ministry of Health and UN Theme Group on HIV/AIDS in China, 2003; Li et al., 2010, 2016).

Data for the current study were drawn from the baseline assessment of the multi-year (2012–2015) evaluation study of *Child-Caregiver-Advocacy-Resilience* (*ChildCARE*), a resilience-based intervention for children affected by parental HIV (Harrison et al., 2017, 2018, 2019; Li et al., 2017). Specifically, researchers worked with anti-epidemic centers in Henan to identify five villages with high HIV prevalence. In collaboration with local school districts and social welfare systems, a list was generated of families caring for children (ages 6–17) affected by parental HIV (i.e., child had ≥1 parent who was living with HIV or who had died an AIDS-related death). Families were randomly invited to participate until the target sample size (i.e., ~800 child-caregiver dyads) was achieved. HIV-positive children were ineligible for the study, with HIV status confirmed by caregivers. Caregivers provided consent for themselves and their child to participate in the longitudinal randomized controlled trial of ChildCARE (Harrison et al., 2017, 2018, 2019; Li et al., 2017). All data from the current study were collected prior to the implementation of any intervention. The study protocol was approved by the Institutional Review Boards at the University of South Carolina, Wayne State University, and Henan University.

### Participants

A total of 790 children affected by parental HIV participated in the current study. Children had a mean age of 10.5 years (*SD* = 1.99), and most (99.2%) were of Han ethnicity. A total of 51.6% (*n* = 408) were male, and 48.3% (*n* = 382) were female. A total of 41.5% of caregivers reported elementary school to be the highest level of education attained. Most caregivers were farmers (32.9% of male caregivers; 46.2% of female caregivers) or migrant workers (42.6% of male caregivers; 28.3% of female caregivers). A majority (52.5%) of caregivers reported a yearly household income of <1,000 yuan (i.e., <$150 USD). The majority of children (72.6%) had one parent living with HIV (*n* = 563), and 15.1% of children had both biological parents living with HIV (*n* = 117). A minority of the samples were orphaned, having lost either one (9.3%, *n* = 72) or both parents (3.1%, *n* = 24) to AIDS-related deaths. Most children had siblings (*M* = 1.77 siblings).

### Measures

#### Demographics

Children reported on a variety of demographic items including age, gender, and parental vital status (i.e., living or deceased).

#### Trauma Exposure

A modified version of the *Lifetime Incidence of Traumatic Events, Student Form* (*LITE-S*; Greenwald and Rubin, 1999) assessed children’s exposure to traumatic events. Children were asked to indicate whether they had experienced 15 separate traumatic events (e.g., car accident and natural disaster). Traumas were summed to create a total trauma exposure score, with higher scores indicating greater exposure.

#### Anxiety

Children’s anxiety was assessed with a 6-item subscale of the *Child Rating Scale* (*CRS*; Hightower et al., 1986). Children reported on recently experienced anxiety symptoms (e.g., feeling nervous, feeling scared, and worrying about mistakes) at school using a 4-point scale. The mean score was calculated, and higher mean scores indicated a greater number of anxiety symptoms. Cronbach’s *α* for the current study was 0.51.

#### Depression

Children’s depressive symptoms were assessed with a 10-item version of the *Center for Epidemiological Studies Depression Scale for Children* (*CES-DC*) previously validated for use among other Chinese populations (Cheng et al., 2006). Children were asked to indicate (1 = rarely or not at all to 4 = all of the time) how often they had recently experienced 10 symptoms of depression, including sadness, sleep disturbances, and loneliness. The mean score was calculated, with a higher mean score indicating more symptoms of depression. Cronbach’s α for the current sample was 0.62.

#### Peer Social Functioning

Children’s peer social functioning was measured with an 8-item subscale from the *Children’s Loneliness and Social Dissatisfaction Scale* (Asher et al., 1984). This subscale assesses perceptions of social adequacy and self-reported satisfaction in social relationships. Sample items included “It’s easy for me to make new friends at school” and “I get along with other kids.” Children indicated their agreement with items using a 4-point scale (1 = does not describe me at all to 4 = totally describes me). Cronbach’s α in the current study was 0.60.

### Procedure

After consent, children completed a baseline survey to collect information on demographics and a range of psychosocial, educational, and physical health variables. Data reported in the current study represent one subset of the comprehensive survey battery. For the baseline assessment, children completed survey questionnaires in a private location at their local schools in the presence of trained research staff. Research staff consisted of graduate students and faculty with significant training in child development that received additional training in age-appropriate data collection. Children were grouped by similar grade level for data collection. A small minority (~2%) of children needed the staff to read items aloud, individually and privately, and to record their oral responses. All children received small gifts following survey completion.

### Analytic Plan

To test the direct effect of trauma exposure on peer social functioning, linear regression analysis was performed in SPSS 24.0. Children’s gender, experience of parental death, and age were included as covariates. To test the mediation model, the SPSS PROCESS macro was used to examine depression and anxiety as two parallel mediators in the association between trauma exposure and peer social functioning. The indirect effect was assessed with bootstrapping method (Preacher and Hayes, 2004). The indirect effect was considered significant if the 95% bias-corrected bootstrap confidence intervals (CIs) did not contain 0 (i.e., based on 5,000 bootstrap samples). Demographic covariates were controlled for mediators and the dependent variable. The PROCESS macro was used to test whether child’s age moderated the paths from trauma exposure to depression and anxiety and the paths from depression and anxiety to peer social functioning. Simple slope analysis was performed to interpret the moderating effect.

## Results

Table 1 displays descriptive information and correlation coefficients between study variables. Significant correlations were found between trauma exposure and peer social functioning (*r* = −0.10, *p* = 0.004). Trauma exposure was also correlated with depression and anxiety (*r* = 0.18, *p* < 0.001; *r* = 0.19, *p* < 0.001, respectively). In addition, depression and anxiety were correlated with peer social functioning (*r* = −0.37, *p* < 0.001; *r* = −0.18, *p* < 0.001, respectively).

**Table 1:** Descriptive statistics and correlations between study variables.

Variables	1	2	3	4	5	6	7
1. Gender	-						
2. Parental death	−0.04	-					
3. Age	−0.08^*^	0.00	-				
4. Trauma exposure	−0.04	0.09^*^	0.01	-			
5. Depression	−0.07	0.04	−0.06	0.18^***^	-		
6. Anxiety	0.04	−0.03	−0.05	0.19^***^	0.25^***^	-	
7. Peer social functioning	0.06	−0.00	0.17^***^	−0.10^**^	−0.37^***^	−0.18^***^	-
*M*	0.48	0.12	10.51	2.53	20.19	1.95	2.79
*SD*	0.50	0.33	1.99	2.18	4.47	0.56	0.52
Cronbach’s alpha	-	-	-	-	0.62	0.51	0.60

Gender was coded as 0 = boy and 1 = girl. Parental death was coded as 0 = no and 1 = yes.

* *p* < 0.05;

** *p* < 0.01;

*** *p* < 0.001.

Linear regression modeling showed that trauma exposure was significantly associated with peer social functioning (*β* = −0.10, *p* = 0.004) and remained significant (*β* = −0.10, *p* = 0.005) when controlling for gender, parental death, and age.

**Figure 1:**
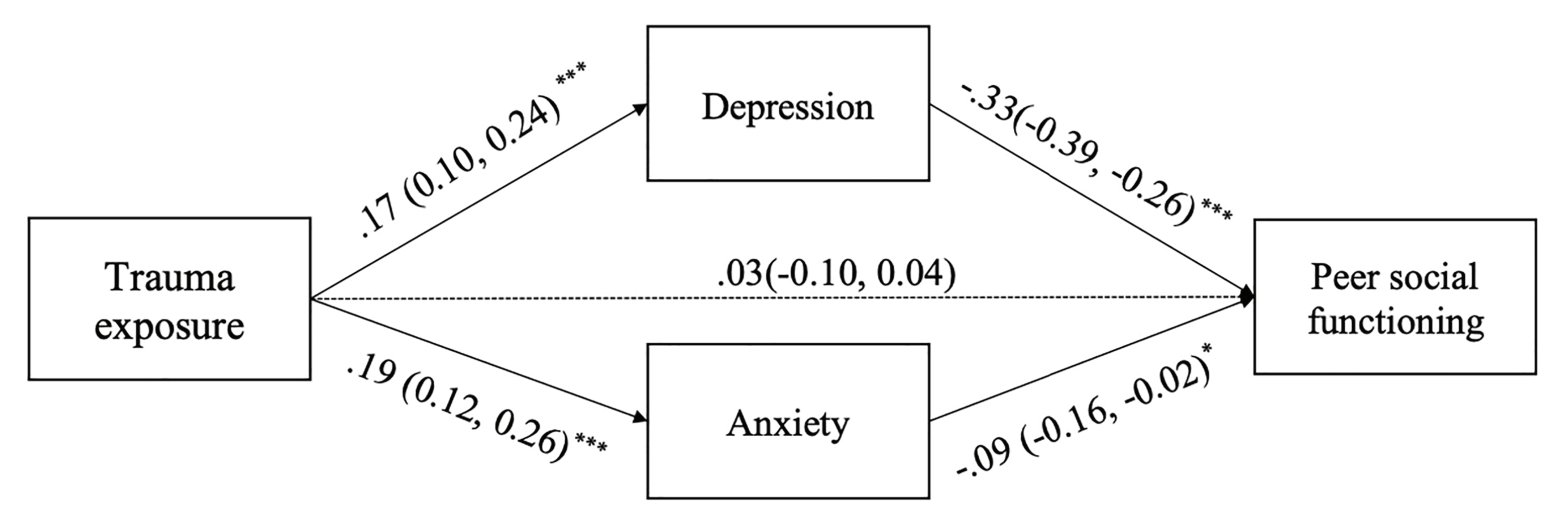
The mediation model of the indirect effect of trauma exposure on peer social functioning *via* depression and anxiety. Standardized coefficients (confidence intervals) were presented. Demographic variables, including gender, parental death, and age, were controlled but not displayed for simplification. There were significant indirect effects from trauma exposure to peer social functioning *via* depression and anxiety (*via* depression = −0.06, 95%CI[−0.09, −0.03]; *via* anxiety = −0.02, 95%CI[−0.04, −0.00]). ^*^p < 0.05; ^***^p < 0.001.

Mediation analyses showed that trauma exposure was related to both depression and anxiety (*β* = 0.18, *p* < 0.001; *β* = 0.19, *p* < 0.001, respectively). Depression and anxiety, in turn, were related to peer social functioning (*β* = −0.34, *p* < 0.001; *β* = −0.09, *p* = 0.011, respectively). The association between trauma exposure and peer social functioning became statistically nonsignificant (*β* = −0.02, *p* = 0.47). There were significant indirect effects from trauma exposure to peer social functioning *via* depression and anxiety (*via* depression = −0.06, 95%CI[−0.09, −0.03]; *via* anxiety = −0.02, 95%CI[−0.04, −0.00]). Controlling for gender, parental death, and age, all mediating path coefficients remained significant (see Figure 1).

**Figure 2:**
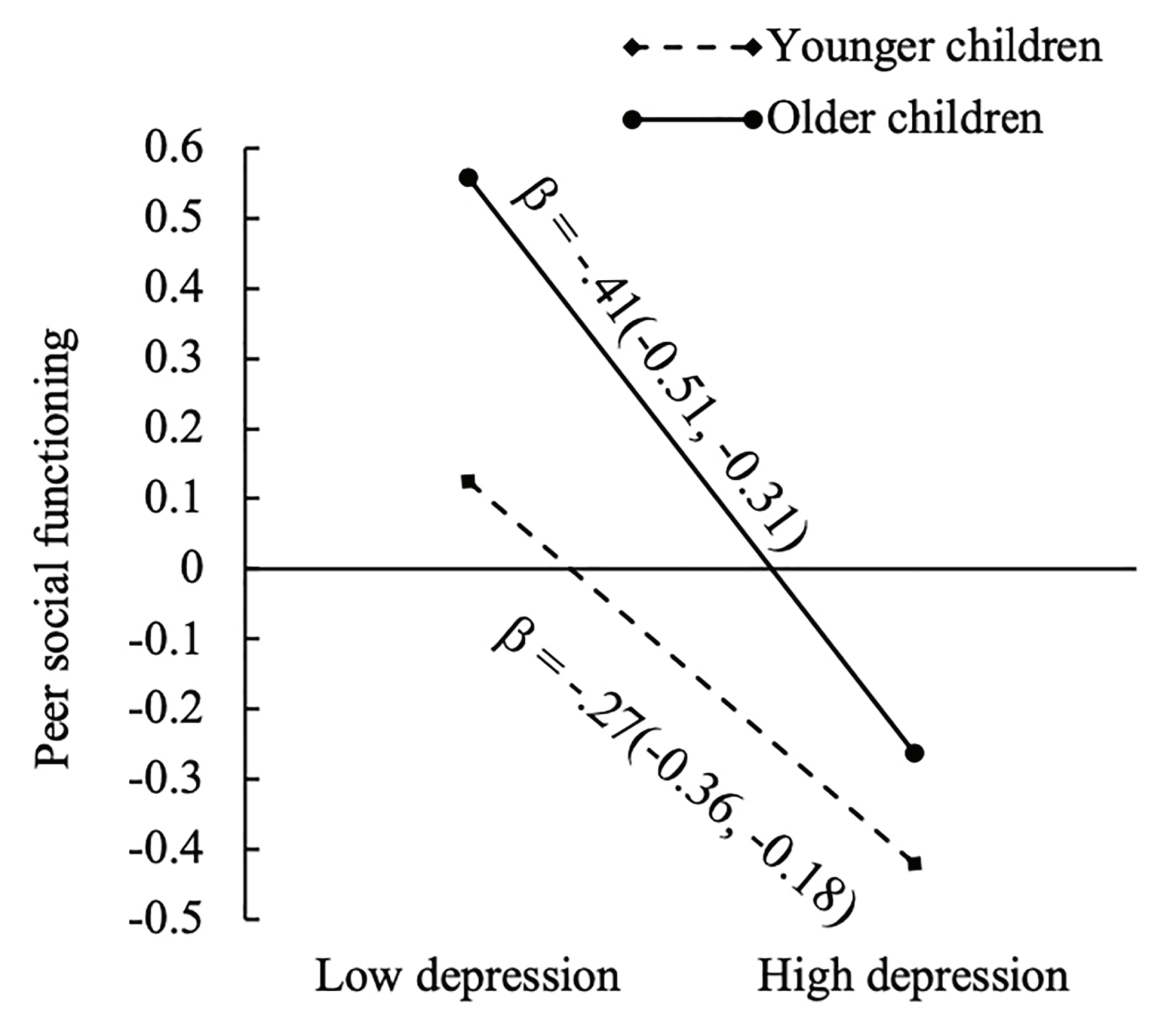
Simple slopes of peer social functioning at different values of depression and age. Standardized coefficients (confidence intervals) for the slopes were presented. High and low levels of depression represented 1 *SD* above and below the mean depression, respectively. Older and younger children represented 1 *SD* above and below the mean age, respectively.

Moderated mediation analyses showed no interactive effects of trauma exposure and age on depression or anxiety (*β* = 0.01, *p* = 0.77; *β* = 0.01, *p* = 0.83, respectively). There was a significant interaction between depression and age on peer social functioning (*β* = −0.07, *p* = 0.047) but not between anxiety and age on peer social functioning (*β* = 0.03, *p* = 0.44). Simple slope analyses showed that the negative association between depression and peer social functioning was stronger in older children (1 *SD* above mean age, *β* = −0.41, *p* < 0.001) than younger children (1 *SD* below mean age, *β* = −0.27, *p* < 0.001; see Figure 2). Conditional indirect effects of trauma on peer social functioning *via* depression were estimated for younger children and older children. Results showed that the conditional indirect effect was significant for younger and older children (*β* = −0.05, 95%CI[−0.09, −0.02]; *β* = −0.08, 95%CI[−0.13, −0.03], respectively). After controlling for gender and parental death, the interacting effect of depression and age on peer social functioning became nonsignificant (*β* = −0.06, *p* = 0.078; see Table 2).

**Table 2:** Moderated mediation regression analysis.

	Depression			Anxiety			Peer social functioning		
Variables	*β*	*CI*	*p*	*β*	*CI*	*p*	*β*	*CI*	*p*
Gender	−0.11	−0.25, 0.03	0.11	0.10	−0.04, 0.24	0.16	0.11	−0.02, 0.24	0.11
Parental death	0.05	−0.17, 0.26	0.68	−0.15	−0.36, 0.07	0.17	0.04	−0.17, 0.24	0.72
Age	−0.06	−0.13, 0.01	0.099	−0.06	−0.13, 0.02	0.13	0.14	0.07, 0.21	<0.001
TE	0.17	0.10, 0.24	<0.001	0.19	0.12, 0.26	<0.001	−0.03	−0.10, 0.04	0.40
TE × Age	0.00	−0.07, 0.07	0.94	−0.01	−0.07, 0.06	0.89	-	-	-
Depression	-	-	-	-	-	-	−0.34	−0.40, −0.27	<0.001
Anxiety	-	-	-	-	-	-	−0.08	−0.15, −0.01	0.018
Depression × Age	-	-	-	-	-	-	−0.06	−0.13, 0.01	0.078
Anxiety × Age	-	-	-	-	-	-	0.02	−0.05, 0.09	0.62
*R*^2^	0.04^***^			0.04^***^			0.17^***^		

Standardized coefficients (*β*) were presented. Gender was coded as 0 = boy and 1 = girl. Parental death was coded as 0 = no and 1 = yes. TE, trauma exposure.

*** *p* < 0.001.

## Discussion

The present study provides insight to the relationship between trauma exposure, mental health, and peer social functioning for children affected by parental HIV. While previous research has shown that such children are at increased risk for ACEs, including parental death and caregiving transitions (Li et al., 2009), the current study demonstrates that trauma exposure is associated with more symptoms of anxiety and depression, as well as poorer peer social functioning for this vulnerable group. Further, mental health symptoms were found to significantly mediate the relationship between trauma exposure and peer functioning. Therefore, ACEs may negatively impact peer social skills through the mechanism of increased symptoms of anxiety and depression. This finding highlights the importance of mental health screening and intervention for HIV-affected children – particularly those with past experiences of trauma. Providing trauma-informed care and evidence-based mental health interventions may be ways to buffer the negative impacts of trauma on peer social functioning.

Despite nearly four decades of the HIV epidemic, mental health interventions for children affected by parental HIV remain in their infancy – particularly in China. Lin et al. (2014) have developed a six-session grief-processing intervention for Chinese children orphaned from AIDS. The intervention includes process-oriented activities to help children cope with parental loss, as well as coping skills activities. Preliminary evidence supports that the intervention reduces trauma-related and depressive symptoms (Lin et al., 2014). However, this intervention was developed specifically for AIDS orphans. Past research has found that children affected by parental HIV are also more likely to experience adversity, including parental separation and/or divorce, removal from their biological family, harsh physical punishment, forced sexual activity, and other violent acts (e.g., robbery; Li et al., 2009). Thus trauma-informed interventions are needed not only for AIDS orphans but also for children affected by parental HIV (Ko et al., 2008). To the best of our knowledge, there are currently no published findings from trauma-focused interventions for this group of vulnerable Chinese children.

A few broader psychosocial interventions have been developed for children affected by parental HIV, including the ChildCARE intervention. ChildCARE is a resilience-based, multi-level intervention for children affected by parental HIV in China. While the intervention has shown preliminary efficacy in improving many child and caregiver outcomes (Harrison et al., 2017, 2018, 2019; Li et al., 2017), the group-based intervention was not designed to treat individual clinical symptoms of trauma, anxiety, or depression. Future modifications could be useful in addressing this gap. For instance, the use of a “triage” approach may be needed, in which eligible youth are screened for mental health symptoms and exposure to ACEs prior to participating in ChildCARE. Low-risk children could receive the standard group intervention delivered by paraprofessionals. In contrast, high-risk children could participate in individual therapy by trained clinicians.

Additional research on culturally appropriate and effective therapies for Chinese children with depression and anxiety is needed generally. While cognitive behavioral therapy (CBT) was introduced in Mainland China in the 1980s, its use is not widespread. In 2017, there were estimated to be <1,000 psychiatrists and psychologists in China (i.e., a nation with ~1.4 billion people; World Bank, 2019) who had systematic training in CBT consistent with established training protocols, with even fewer having actual experience utilizing CBT with children (Liu et al., 2017). Given that CBT is the first-line clinical treatment for anxiety and depression in children and adolescents from Western cultural contexts (Compton et al., 2004), it is essential to understand whether CBT is effective for Chinese children. Recently, the first meta-analytic study was conducted to examine efficacy of CBT among Chinese populations (Ng and Wong, 2018). The findings supported that CBT is an efficacious therapeutic approach for Chinese clients – particularly for anxiety and depression, yet only three of the 55 included studies of CBT efficacy among youth in Mainland China (Ng and Wong, 2018). Greater efforts are needed to develop and/or adapt culturally-tailored, theory-based psychological treatments for Chinese youth, as well as to evaluate their feasibility, acceptability, effectiveness, and implementation.

Current findings also highlight the understudied area of peer social functioning for youth affected by parental HIV. Among general child populations, good social skills are linked with academic success and peer support, and deficits in social functioning are associated with internalizing and externalizing problems, as well as academic difficulties (Catalano and Hawkins, 1996; Elliott et al., 2001; Ray et al., 2017). Competent social skills and positive interpersonal relationships are thus important for all children but may be particularly salient protective factors for vulnerable youth. Previous studies have identified associations between perceived social support and psychological wellbeing for children affected by parental HIV in China using cross-sectional and longitudinal designs (Hong et al., 2010; Mo et al., 2014; Qiao et al., 2014). The current study expands this work by moving from the broad domain of perceived social support (e.g., from family, friends, teachers, and significant others) to the narrower construct of peer social functioning, with findings indicating that peer social skills are negatively associated with internalizing symptoms, as well as past exposure to trauma.

Psychosocial interventions for youth affected by parental HIV are often delivered in peer group settings; however, enhancing peer social skills is rarely a direct aim of such interventions (Betancourt et al., 2013; Skeen et al., 2017). A variety of cognitive-behavioral and social learning-based strategies are effective in building social skills of children and adolescents (Park et al., 2017), including behavioral and video modeling, and self-management strategies. Incorporating these strategies into existing psychosocial interventions for HIV-affected youth may be an efficient way to strengthen social skills and improve peer relationships. For instance, the ChildCARE intervention (Harrison et al., 2017, 2018, 2019; Li et al., 2017) delivers psychosocial programming in small, naturally occurring peer groups, which are an opportune environment for youth to practice positive social skills, engage in problem-solving around friendship challenges, and observe positive skills modeled by peers and facilitators. Any such intervention should also address HIV-related stigma, due to the wide-ranging impacts of stigma and discrimination on children’s peer relationships.

The present study also found that the more depressed a child reported feeling, the lower their social functioning. This relationship was significant for both younger and older children but showed a stronger association in the older sample. According to the stress sensitization hypothesis, children who experience early trauma can develop maladaptive stress responses, which can lead to internalizing problems and even PTSD later in development (Hammen et al., 2000; McLaughlin et al., 2010; Ogle et al., 2013). As children age, they may also experience new traumas and be impacted by the compounding effects of multiple traumas. Children affected by parental HIV may be at an increased risk for depression and social deficits as they age, further highlighting the need for early and prompt intervention.

### Limitations

Although the present study included a large sample of children affected by parental HIV, there are several limitations. The study relied on brief, self-reported screening measures, as opposed to comprehensive diagnostic instruments or clinical interviews. This impacts the ability to generalize findings to clinical samples. Further, the use of brief measures may contribute to the low Cronbach’s alphas seen in several of the scales, since internal reliability is based on the size of the correlation, as well as the number of items (Streiner and Norman, 1989). Future studies may wish to include expanded measures, as well as parent- and teacher-reported data that could provide a more holistic view of the child’s functioning. Additionally, measures used in the study were not developed specifically for children impacted by parental HIV, and thus could fail to capture symptoms and experiences unique to this population.

Another limitation is the use of cross-sectional data rather than longitudinal data that would have allowed us to demonstrate causal relationships. In future studies, temporal data could provide insight into the development of internalizing symptoms and their mechanistic role in the relationship between ACEs and social dysfunction. Additionally, data on the timing of trauma exposures were not collected. Some evidence suggests that the age at which trauma is experienced impacts outcomes for children and adolescents; future studies should investigate how the timing of ACEs is associated with later mental health and peer functioning. The sample also included a relatively broad age range of children. Symptoms of anxiety and depression, as well as recall of past traumatic events, may vary across development. Thus, future studies may wish to restrict their sample to tighter ranges.

## Conclusion

Children with parental HIV are at a heightened risk for a variety of negative outcomes. This study demonstrates that trauma exposure for this group is associated with greater symptoms of anxiety and depression, as well as poorer peer functioning. The present study is among the first to link early trauma exposure to peer social functioning for this group, and it demonstrates that anxiety and depression mediate this relationship between trauma exposure and peer functioning. Findings support the need for interventions that target social skills of children made vulnerable by HIV. Furthermore, screening for trauma exposure and implementing trauma-informed programming for affected youth may be important ways to ensure that trauma-related symptoms are recognized and addressed promptly.

## Data Availability Statement

The raw data supporting the conclusions of this article will be made available by the authors, without undue reservation.

## Ethics Statement

The studies involving human participants were reviewed and approved by the Henan University Institutional Review Board, the University of South Carolina Institutional Review Board, and the Wayne State University Institutional Review Board. Written informed consent to participate in this study was provided by the participants’ legal guardian/next of kin.

## Author Contributions

XL served as principal investigator of the parent study. JE and SH conceptualized the paper, drafted the remainder of the paper, and finalized the paper. YJ developed the analytic plan, executed the statistical analysis, and drafted the Results section. All authors provided feedback to early drafts. All authors contributed to the article and approved the submitted version.

### Conflict of Interest

The authors declare that the research was conducted in the absence of any commercial or financial relationships that could be construed as a potential conflict of interest.
